# “I’m Afraid If This Goes Wrong… What Will Become of Me?”: The Psychological Experience of Grandparents in Pediatric Palliative Care

**DOI:** 10.3390/healthcare11172391

**Published:** 2023-08-25

**Authors:** Alexandra Jóni Nogueira, Maria Teresa Ribeiro

**Affiliations:** CICPSI, Faculty of Psychology, University of Lisbon, Alameda da Universidade, 1649-013 Lisbon, Portugal; mteresaribeiro@psicologia.ulisboa.pt

**Keywords:** pediatric palliative care, life-limiting conditions, grandparents, family, psychological experience

## Abstract

Portugal has been identified as the European country with the most rapid evolution of Pediatric Palliative Care provision, where approximately 7800 children have life-limiting conditions. This is a highly complex experience not only for the children and their parental caregivers, but also for their healthy siblings and grandparents. The present descriptive-exploratory study seeks to contribute to the understanding of the psychological experience of life-limiting conditions in grandparents. A total of 19 families, consisting of 15 grandmothers and 4 grandfathers, completed a sociodemographic and clinical data sheet and a semi-structured interview was conducted in which they shared their testimony. The results of the thematic analysis highlighted an integrated view on 10 important dimensions in the grandparental experience and promoted creative responses by means of their own perspective. However, it has some limitations, such as the small sample size and the data collection procedure via telephone. The results contribute to the design of specific intervention methodologies in an ecosystemic approach and suggest further research to explore more protective factors and communication with health professionals. For psychological intervention, it is suggested considering the identification of individual and family resources that contribute to the activation of key processes in resilience and posttraumatic growth.

## 1. Introduction

Pediatric palliative care (PPC) is provided to children and adolescents who will not get better from their life-limiting conditions, regardless of their diagnosis or stage of illness [1,2]. It constitutes a basic human right, recognized by the World Health Organization, the main goal of which is to promote the child’s physical, psychological and spiritual well-being, also ensuring support for the whole family [3]. While provision of this care has expanded on a worldwide scale, Portugal has shown evidence of a more rapid evolution, being positioned at level four (of five) since 2018, i.e., with evidence of broad palliative care provision for children, based on a comprehensive approach of full integration in the health services as well as national policies to support children’s palliative care [4]. In 2018, an estimated 7828 children/youths were identified as having palliative needs [5], which means that they have one of four types of life-threatening illnesses: conditions for which curative treatment may be feasible but can fail; conditions under which premature death is inevitable, potentially involving long periods of intensive illness-oriented treatment; progressive conditions without curative treatment options; and irreversible but non-progressive conditions causing severe disability leading to susceptibility to health complications and the likelihood of premature death [6].

The literature is consensual regarding the impact of a child’s potentially fatal illness on the whole family process, structure and dynamics [7,8]. Therefore, it is crucial to understand the psychological experience of the illness at all levels of the family subsystem, namely the parental caregivers, the healthy siblings and the grandparents [9]. This comprehensive approach can inform health professionals for their intervention with families in the context of this stressful life event as it helps in the alignment of their real needs and challenges with the resources provided by the multidisciplinary team, within an ecosystemic approach [10].

Grandparents play a central role in the family sphere and are increasingly present in health care settings, although their psychological experience has been underexplored. It is therefore essential to develop specific studies and methodologies to understand the role, responsibilities, and challenges of grandparents in PPC [11]. The psychological experience in this context is understood as the set of emotions, thoughts and behaviors originating from and/or experienced within the context of the illness.

In the Western culture, becoming a grandmother/grandfather may symbolize a special status and be considered a promoting factor in the perception of life satisfaction [12]. In addition, children with a complex chronic illness have a higher life expectancy nowadays due to medical and technological advances, as do the elderly, which favors a longer intergenerational relationship over time [11]. Thus, PPC teams need to be aware of the generational impact of a life-limiting condition at a pediatric age and acknowledge the role of grandparents as being complex and dependent on their identity and the reorganizations, idiosyncrasies and needs of each family [13].

A study by Miller, Buys and Woodbridge of Australian grandparents of children with physical and/or intellectual disabilities highlighted their need to be available for their grandchildren and for their own children [14]. As the primary internal support resource in the family, they tend to self-sacrifice, prioritizing family needs, mediating relationships, their concerns about the future, and their choice to remain positive. The literature suggests that the complexity of support and tasks performed by grandparents in the family may be of a guiding, playful, financial, instrumental, socio-emotional and spiritual nature [15,16]. Their investment is mainly determined by relational and geographical factors. According to Dunifon, Near and Ziol-Guest, physical distance appears to have a negative impact on the quality of the grandparent–grandchild relationship, especially when they are more than 100 miles away. Nevertheless, in light of the increased use of technological platforms and video calls, greater emotional proximity is possible despite the physical distance [13]. 

Other studies [17] also point to the importance of the mediation of the intermediate generations (children and their partners) in the quality of the grandparent–grandchild relationship. This is particularly important in family reorganizations such as divorce, where grandchildren with an illness are left under the responsibility of a parent who is not a child of the grandparent in question, as contact is reduced [18]. In 2018, Tan highlighted that adolescent grandchildren’s adjustment also depends on secure emotional connections with grandparents, which contribute to relief from the cumulative stress of adverse life events [19].

Research has focused on grandparents’ experiences from a mostly retrospective perspective, in the context of mourning following the death of a grandchild. Nevertheless, it is known that grandparents try to maintain the provision of emotional support to their child’s caregivers and their grandchildren, while they do not always have room to express their own right to grieve. The repression of psychological distress results from an effort to protect the family and serve as an “anchor” for all its elements, especially the children who are experiencing such adverse and demanding life circumstances [11,14]. Coupled with emotional inhibition is the attempt to express positivity, which may lead to the accumulation of muted difficulties and frustrations [20]. This family subsystem feels they must constantly be a source of support for their children, their sick grandchild and healthy grandchildren, thus giving rise to a “triple concern” [16,21].

Despite their involvement in the care of grandchildren with palliative care needs, grandparents do not always feel integrated into the multidisciplinary care approach. In the context of family disorganization and adversity, hospital and institutional teams need to consider the grandparents’ suffering and take an active approach towards them, acknowledging their concerns and anguish, while promoting support for their emotional needs, from the moment the illness is diagnosed [22]. This accompaniment for multigenerational families also translates into grandparents’ better adaptation to the illness, as well as a more harmonious integration of the grieving process when the sick grandchild dies [23,24]. Given the generational position of grandparents, their grief is strongly influenced by exposure to the emotional pain of their spouses, children, and healthy grandchildren [25].

It is therefore critical to validate their grief, assist in loss management, promote intra-family cohesion and solidarity, as well as to refer the grandparents to the available resources (e.g., handouts, support from mental health professionals, contact with families in similar circumstances) [26]. Bibliotherapy has been found to be effective in promoting more adaptive coping, especially at the end of grandchildren’s lives [27].

Finally, this study seeks to understand the experience of the grandparent subsystem—with grandmothers and grandfathers—from their own perspective and considering the heterogeneity of the existing complex chronic diseases, thus filling a gap in the scientific research and contributing to the development of systemic psychological intervention in PPC.

Based on a descriptive-exploratory approach, the aim of this study is to contribute to an understanding of the psychological experience of life-limiting conditions in grandparents through their own perspective. Therefore, the guiding research question of this study is as follows: “How do grandparents psychologically experience a life-limiting condition?”. As specific objectives, this study explores: representation of the illness; representation of the sick grandchild; changes in routine and life; family impact; grandparents’ contributions to the family system; social support and coping strategies; emotional impact; “triple concern”; needs identification; and posttraumatic growth.

## 2. Materials and Methods

### 2.1. Procedure

This study is based on a post-positivist and constructivist paradigm. It uses a qualitative, descriptive-exploratory and cross-sectional design. The assessment of the ethical conditions of the study was guaranteed by the Comissão Especializada de Deontologia do Conselho Científico [Board of the Ethical and Deontological Committee] of the Faculty of Psychology of the University of Lisbon.

The evaluation protocol was initially made available online through the GoogleDocs platform, but due to the difficulty in collecting the data, it was applied by telephone. This research was disseminated on social networks, and a non-probabilistic, namely accidental, sampling method was adopted. Simultaneously, several national institutions were contacted with a view to its dissemination and the following entities collaborated in the research: Associação Portuguesa de Osteogénese Imperfeita [Portuguese Osteogenesis Imperfecta Association] [APOI], Associação de Paralisia Cerebral de Lisboa [Lisbon Cerebral Palsy Association] [APCL] and the Sistema Nacional de Intervenção Precoce na Infância [National Early Childhood Intervention System [SNIPI].

The inclusion and exclusion criteria were defined for the recruitment and selection of the sample. Thus, to participate in this study, all the participants were required to be the grandfather or grandmother of a child or youth with a life-limiting condition [6] who had been diagnosed at least 12 months ago. In addition, the child/youth with the condition had to be 18 years of age or younger and the participant had to be an active participant in the family nucleus. Being the primary caregiver of the grandchild and having more than one grandchild with a complex chronic illness were considered exclusion criteria. There was no control group in this study. All the participants gave their verbal informed consent before the interview and then voluntarily participated in this study.

The contact with the families and the respective data collection were carried out by only one researcher, by telephone, aiming at the standardization of procedures and a greater control of external variables. Permission was requested from all participants to make an audio recording of their interview. Subsequently, the telephone interviews were transcribed in their entirety.

Finally, a thematic analysis of the various narratives was performed through an inductive–deductive process, which allowed for the comparison and integration of the results [28]. Initially, the researchers familiarized themselves with the information written and made notes of the ideas related to themes that could become categories. Later, and in a systematic manner, all the answers were coded, placing the relevant data in each defined category and subcategory. The identification of themes occurred mainly at the latent level, in order to identify the underlying conceptualizations. Similarly, the thematic analysis was inductive, since the identified themes were strongly related to the explicit data in the participants’ responses. This process was conducted by the two researchers for being a useful way to identify codes that are not sufficiently well defined, thus producing greater conceptual clarity and strengthening the analysis.

NVIVO^®^ 12 software was used for this analysis, which enables better organization of the data, namely through the category tree. For the sociodemographic characterization of the families, SPSS^®^ 29 statistical software was used.

### 2.2. Participants

#### 2.2.1. Grandparents

The sample of this study consists of a total of 19 grandparents of children in PPC, with 15 grandmothers and 4 grandfathers. They are aged between 44 and 78 years (Mdn = 63.00, IQR = 12) and are all of Portuguese nationality, except one who is Brazilian. As regards to marital status, 14 (73.7%) are married and 5 (26.3%) are widowed. The districts of Porto (n = 4), Faro (n = 3), Lisbon (n = 3), Aveiro (n = 2), Guarda (n = 2), Setúbal (n = 1), Braga (n = 1), Santarém (n = 1), Coimbra (n = 1) and Castelo Branco (n = 1) are represented as the location of residence. Regarding academic qualifications, five grandparents have completed between the 1st and 4th grade, seven between the 5th and 9th grade, four have completed the 12th grade and three hold a bachelor’s degree. As for their current employment situation, nine (47.4%) are retired, four (21.1%) are employed, four (21.1%) are unemployed and two (10.5%) are on leave of absence. Of the 19 participants, 7 (36.8%) have used mental health services in the past and 5 (26.3%) currently benefit from these services. As for their physical health, 15 grandparents (78.9%) have identified problems such as hypertension, thyroid and heart problems, kidney failure and rheumatological problems.

#### 2.2.2. Children with Life-Limiting Conditions

There are 12 male children (63.2%) and 7 female children (36.8%), with ages ranging from 1 to 16 years (Mdn = 9.00, IQR = 6). All the diagnostic categories in PPC (TFSL, 2018) are represented in this study, with particular emphasis on some conditions, such as cerebral palsy, cystic fibrosis, FIRES syndrome, Costello syndrome, Treacher Collins syndrome, neutropenia and neuroaxonal dystrophy. Of these children, four (21.1%) are dependent on medical respiratory technology and all have been subjected to hospitalizations. Nine (47.4%) grandparents have known the diagnosis of the disease for the past one to five years, five (26.3%) for the past five to nine years and five (26.3%) grandparents have known for the past nine to seventeen years. In most of the participating families, the mothers (94.7%) are considered the main caregiver and the grandmothers (47.4%), fathers (10.5%) and the grandparents with fathers (26.3%) are the secondary caregivers. As for knowledge regarding PPC, 16 of the grandparents (84.2%) are not familiar with this service while 3 (15.8%) are.

#### 2.2.3. Family Household

The grandparents’ households are composed of between one and six members (Mdn = 2.00, IQR = 1) and in nine situations (47.4%), they live with their partner while five grandparents (26.3%) live with their partner and relatives. The sick grandchildren’s households are composed of between two and five members (Mdn = 4.00, IQR = 1). The father is part of the household in 11 families (57.9%) and there are healthy siblings in 11 families (57.9%). Regarding the geographical distance, four (21.1%) grandparents of this study live with their children and grandchildren, eight (42.1%) grandparents live within 6 miles of their grandchildren, three (15.8%) live within 186 miles and one (5.3%) lives in another country.

### 2.3. Instruments

The protocol for assessing the psychological experience of illness by grandparents consists of a sociodemographic and clinical data sheet and a structured interview script.

#### 2.3.1. Sociodemographic and Clinical Data Sheet

The aim of this sheet is to collect information to characterize the study sample, namely data regarding the grandparents (e.g., age, employment status and physical health), the grandchild with a life-limiting illness (e.g., age, illness diagnosis and technological dependence) and the family (e.g., own and grandchildren’s household composition and geographical distance).

#### 2.3.2. Structured Interview Script

This script followed an incomplete narrative based on sharing a testimony. The dimensions under assessment correspond to the specific features of this population’s process of adaptation to the illness, and were selected following the literature review, namely: representation of the illness; representation of the sick grandchild; changes in routine and life; family impact; grandparents’ contributions to the family system; social support and coping strategies; emotional impact; “triple concern”; needs identification; and posttraumatic growth. This script was constructed based on Narrative Therapy strategies [29], where some guiding questions are asked and the content of each participant’s answers reflects their dominant narrative about the way they experience the illness [30], such as: “If your family were to give you a trophy for ‘best grandmother/grandfather’, what three characteristics do you think they would highlight?” and “What interesting discoveries have you made about yourself and your family?”. The interview is estimated to last an average of 45 min.

## 3. Results

The data analysis resulted in a system of categories, with a total of 105 items, of which 10 were the main categories that aimed to meet the specific objectives of this study.

### 3.1. Representation of the Illness

In the present research, 9 of the 19 grandparents displayed specific knowledge about the disease, the care required and the particularities of the child/youth. On the other hand, six grandparents were found to have less precise knowledge, sometimes associated with the desire for a cure or miracles. A further seven grandparents emphasized their desire to see their sick grandchild improve and conquer achievements. 

The majority of the sample reported the constant nature of the care and supervision provided to the sick child/young person: “*It’s very difficult, in 15 s things can happen… we are always afraid and have to be vigilant constantly. It’s his mother who takes care of him 24 h, day and night!*” (A., maternal grandmother of a 16-year-old with cerebral palsy). Also, 10 grandmothers focused on the limitations resulting from the disease at the family, functional and emotional levels: *I don’t know what future she has, she is very angry, it’s uncomfortable for her… she can’t play freely, she has little balance (…) always falling and always covered in bandages!* (M., maternal grandmother of a 9-year-old child with deletion syndrome 1p36).

It should also be noted that 17 of the 19 grandparents mentioned attempts to attribute some normality to the circumstances, seeking not to distinguish between their sick and healthy grandchildren, to deconstruct society’s prejudices and value judgments, and also to compensate for the child’s functional limitations by focusing on their other skills: *It’s always going to be for life…the parents constantly worried…but I treat him like I treat his sister, normally…like any child without a serious illness!* (A., paternal grandfather of a 9-year-old child with Costello syndrome). 

### 3.2. Representation of the Sick Grandchild

In relation to how grandparents perceived and characterized their grandchildren, most of them referred to great emotional proximity: *I love my grandchildren very much, I like playing with them, putting them at the same level, talking to them…and I’m really enjoying being a grandparent! We have more time right now, we understand things better now than when we were parents. We see life differently, being a grandparent is wonderful!* (M., maternal grandfather of an 11-year-old child with profound cerebral palsy). In line with this finding, 17 grandparents also highlighted the role of the unconditional love they feel: *Look, for me, it’s all love, he’s a golden boy! My little boy is golden, he’s what I live for. I’m not kidding, he’s an angel in my life and he makes me happy! He doesn’t laugh these days, but his laughter means the world to me!* (I., paternal grandmother of a 7-year-old child with neuroaxonal dystrophy).

In further questions on their representation of their grandchildren, 8 grandparents mentioned that they transmit a lot of happiness, and 14 grandparents reinforced the resilience of the children/youths, admiring their courage and strength: *He has unconditionally resisted everything!* (A., maternal grandmother of a 4-year-old child with FIRES Syndrome).

### 3.3. Changes in Routine and Life

In this regard, 12 of the 19 grandparents highlighted the need to adapt their personal commitments, often depriving themselves of social, leisure and self-care activities: *I retired at 55, I thought I would do a lot of things in my life, volunteering, everything… I have become more limited than when I was working all that time and now even more so since they got divorced… my life has been turned upside down completely!* (M., maternal grandfather of an 11-year-old child with profound cerebral palsy). Additionally, 12 grandparents also mentioned the care required due to the COVID-19 pandemic, as illustrated in this quote: *Sometimes months go by and I don’t see D. or I’m not with him because of the pandemic and also because my professional activity forces me to be in contact with lots of people and I’m afraid of transmitting the virus to him…* (J., great uncle of a 5-year-old child with hypoxic-ischemic encephalopathy with epilepsy).

However, and although with less expression, some grandparents highlighted the child’s accompaniment to therapies and during hospitalizations, as well as the lack of attention to their spouses and other children.

### 3.4. Family Impact

In this study, all the grandparents claimed to have seen an increase in the mutual aid, knowledge and sharing of affections in the family sphere: *Essentially, the basis is a lot of love, because when there is love, you are getting to know the child at your side! I know he doesn’t talk, but I know when he has a tummy ache. Both C. [daughter] and I are able to identify all this (…) almost! The almost part is lacking, as if God is saying: ‘This is for you to find out!’. I know it’s crazy, but that’s the way I see it*. (M., maternal grandmother of an 11-year-old child with cerebral palsy). Moreover, 10 grandparents reported regular communication in the family: *Before our concerns were with our daughter, now it’s the boy… always, always, always thinking about the boy! (…) We talk to them every day by WhatsApp so that we can see each other and spend a little bit of time with him*! (A., maternal grandmother of a 5-year-old child with hypoxic-ischemic encephalopathy with epilepsy).

In the context of family dynamics, seven grandparents highlighted their awareness of their grandchildren’s sibling rivalry, although mentioning that some of the tension stems from jealousy: “*Even though they say V is my favorite., I love them all, they are all lovely!*” (A., paternal grandmother of a 16-year-old girl, with Laparoschisis with intestinal transplantation) and *My granddaughter has accepted her brother very well! (…) she was 5 years old at the time (…) she used to say, “my brother is not like the others, he doesn’t come with me to the merry-go-round”, then we started to explain that he had an illness (…) and she is a mother hen to her brother! (…) Playing as if he didn’t have any problems, this alone is good as far as we’re concerned!* (A., paternal grandfather of a 9-year-old child with Costello Syndrome).

### 3.5. Grandparents’ Contribution to the Family System

Most grandparents provide direct support to the sick grandchild [14]: *Without the cell phone I get there with toys, I have to have time to play because otherwise he gets fed up… I sing, read a book, some childhood songs, I dance, and he laughs!* (G., maternal grandmother of a 7-year-old child with centronuclear myopathy due to myotubularin 1 deficiency). In addition, 10 grandparents reported supporting their healthy grandchildren: *Wherever I go with her, she talks, she can already say ‘truck’ correctly, and I am so proud because I taught her to say that*! (I., paternal grandmother of a 7-year-old child with neuroaxonal dystrophy).

The grandparents in the present study provide instrumental support in everyday life, helping their children financially, and sometimes staying with their grandchildren temporarily. 

It is also worth noting that 10 grandparents reported providing emotional support to their children and their spouses: 

*He had a complicated phase when she [daughter] would say ‘If D. dies, I’ll die with him, you’ll have to take care of L. [healthy grandchild], promise me that.’ That was the hardest thing for me… because I was also going through the same thing, otherwise I lose a grandson and a daughter! It’s not selfishness, we have to fight for him, because none of this is his fault. If I die, I won’t promise you that, because we need you and L. needs your support. I’ll help you take care of L. and I’ll pick you up if you go down, but you can’t give up*. (F., maternal grandmother of a 4-year-old child with congenital transposition of great vessels, global developmental delay and autism).

Finally, five grandparents mentioned having limitations in their physical health that prevented them from helping the whole family more.

### 3.6. Social Support and Coping Strategies

Grandparents use cognitive, behavioral, emotional, spiritual and social strategies in their attempt to adapt harmoniously to their grandchildren’s illness process. With regard to cognitive strategies, 14 of the grandparents reported trying to increase their knowledge and understanding of the illness, considering the guidance from the professionals and their children, with whom they constantly share information.

As for behavioral strategies, grandparents implement self-care practices and strive to “live one day at a time,” as exemplified in the following quote: *Look, first of all don’t get too caught up about tomorrow, enjoy today, enjoy the small joys and value them, because the really big ones may never come and we’ll lose the small ones…* (G., maternal grandmother of a 7-year-old child with centronuclear myopathy due to myotubularin 1 deficiency).

Among the emotional strategies, most grandparents emphasized the importance of accepting the illness and optimism: *I pride myself on being patient and accepting things as they are*! (A., maternal grandfather of a 9-year-old child with 1p36 deletion syndrome). Regarding spiritual strategies, nine grandparents referred to their religious beliefs as being fundamental, and six grandparents expressed the importance of having faith.

As for social support, 13 of the participants highlighted their spouse’s support as being essential to their quality of life: *My husband is, I don’t know, a wonderful person, he supports me in everything, we support each other. We’ve been married for 46 years*! (A., maternal grandmother of a 4-year-old child with FIRES Syndrome). Support from their children, siblings, friends and the community was also mentioned, as well as the use of video calling.

### 3.7. Emotional Impact

During their adaptation to the implicit circumstances and difficulties of the illness, grandparents’ experience tends to be characterized by weariness, disbelief, successive questioning, sadness and anger: *Sometimes we are capable of not showing it, but inside we are always in pain… inside, right? And we suffer a lot inside*. (A., maternal grandmother of a 16-year-old with cerebral palsy). 

Furthermore, 12 grandparents expressed fear of the future and appear to struggle to verbalize the process of dying and death: *I’m afraid if this goes wrong… what will become of me?* (I., paternal grandmother of a 7-year-old child with neuroaxonal dystrophy). The grandparents also reported being afraid in case they and their children die before their sick grandchild, due to a feeling of leaving the child helpless: *I worry because I don’t know what the future holds, I won’t last forever, my daughter won’t be here forever… will we be able to help him?* (M., maternal grandmother of an 11-year-old child with cerebral palsy). 

In this context, 16 grandparents described their constant worry about the sick child/youth and their caregivers, involving sadness, feelings of helplessness, and attempts to protect their grandchildren. However, 11 grandparents expressed joy at their grandchildren’s achievements and idiosyncrasies, and 6 grandparents expressed frustration with health care professionals.

### 3.8. “Triple Concern”

All the grandparents in this study emphasized their solid and unconditional support for their children and grandchildren: *Whatever I can! Always! Even if I can’t do it for myself, I always have to do it for them and for my other two daughters*. (P., maternal grandmother of 1-year-old child with Treacher Collins Syndrome). In this context, 12 grandparents also reported being totally available for their children and grandchildren, considering them a priority.

The grandparents in this study highlighted feeling constant distress at seeing their children in these circumstances characterized by care provision and suffering, seeking to alleviate their work overload, as in the following example: *I take some things off her hands and do them myself, like the laundry, ironing… yes, I do it myself so as not to overburden her, because she has to be around him 24 h a day…*” (P., maternal grandmother of a 1-year-old child with Treacher Collins Syndrome).

### 3.9. Needs Identification

It is essential to promote opportunities for grandparents to express their experiences: *I think that by not talking, not saying what we think, is much worse for us… far worse, even for our minds!* (P., maternal grandmother of a 1-year-old child with Treacher Collins Syndrome). 

Moreover, six of the interviewed grandparents admitted needing more social support and seven mentioned needing help to manage the anticipatory grief process—“*It’s huge anxiety, life will change completely. I’m afraid something will happen when I call, and my daughter doesn’t answer! We have no peace in our life! (…) always afraid that something more serious will happen…”* (A., maternal grandmother of a 16-year-old girl with cerebral palsy).

### 3.10. Posttraumatic Growth

Regarding the positive psychological change that occurs during or following a difficult illness adaptation process, 14 grandparents highlighted the experience with children vs. grandchildren as being different, enjoying quality time, enjoying the novelty of being grandparents and realizing the influence their parenting role has had on the development of skills: *Playing the grandmother’s role, grandma’s food, even if it’s blended and given through a tube… it’s that grandmother thing!* (J., maternal grandmother of a 3-year-old child with spinal muscular atrophy). In addition, 10 grandmothers reported feeling proud of their grandchildren and their family, as illustrated in the following lines: *I feel proud, I never thought I would go through this, but it happened. It happened, now it is easier to deal with! I am a grandmother of a special child*! (L., maternal grandmother of a 7-year-old child with neuroaxonal dystrophy).

A total of 15 of the 19 grandparents reported feeling personal fulfillment associated with gratification and joy, resulting from an increased perception of competence in the role of grandparents and parents. At the same time, 13 grandparents expressed admiration for the resilience of their children and their spouses.

In the relational scope, most of the grandparents reported the development of intra-family solidarity, as well as a greater willingness to help and an increased ability to feel happiness: “*We have grown in everything, in dedication, love, care, passion and love for N.! She is the ‘youngest’ in the family, everyone is in love with her (…) At first it was a shock, heartbreak that was transformed into love and affection! And into union!*” (J., maternal grandmother of a 3-year-old child with spinal muscular atrophy).

It is also noteworthy that eight grandparents learned to value health and life. Some grandparents also highlighted taking inspiration from their grandchildren’s strength and feeling admiration, mentioning that such perseverance motivated them to fight on a daily basis: *But R. taught me that nothing is impossible. They gave him hours to live when all this happened, the doctor came to me and said that R. had perhaps only minutes to live… and I left the hospital and thought ‘he won’t have minutes to live, my grandson will stay here and show that nothing is impossible’ and he is here, strong and beautiful*! (P., maternal grandmother of a 1-year-old child with Treacher Collins Syndrome).

## 4. Discussion

The grandparents tend to manifest the desire for miracles for the grandchildren’s disease, which represents a more unrealistic dimension in managing the diagnosis. The findings of this study reinforce the importance of grandparents receiving more information about the disease and increasing their understanding of the associated care [31], seeking to enhance their support to the family.

Despite all limitations resulting from the disease, particularly at the functional and emotional levels [32], the grandparents attempt to attribute some normality to the circumstances [33]. In fact, after the diagnosis of their grandchildren’s complex chronic disease, grandparents experience several changes in their lifestyle [34,35], which was also observed in this study.

The emotional proximity with the sharing of significant moments and expressions of affection, mentioned by the participants was also highlighted in the study of Moules and colleagues [34]. The unconditional love they feel is one of their main resources [11].

About the family, the literature focuses on the lack of attention from grandparents to their spouses and other children, which is also found in the present study [34]. Moreover, in the context of the family dynamics, authors Ravindran and Rempel also corroborate the awareness of the grandchildren’s sibling rivalry, among grandparents of children with congenital heart disease [35].

Regarding to their contribution to the family system, the grandparents provide direct support to the sick grandchild, both in terms of care provision and play and being attentive to their needs [14]. They also provide support to their healthy grandchildren [35], to their children and spouses [36], and corroboration can be found in the literature [11].

In addition, Trindade and colleagues explored the family role of grandparents and concluded that grandparents collaborate in health care and expenses, the emotional expression of family members, caring for healthy grandchildren, and promoting respite for the sick child’s caregivers [16]. The grandparents in the present study corroborate these findings, although they have physical limitations [37]. The literature also mentions other factors that influence their ability to be supportive, namely: grandparents’ age, level of schooling and understanding of the disability, family conflicts, stage of the illness, cultural and social environment [16].

From a systemic point of view, the grandparents manifest unconditional support for their children and grandchildren, associated with a desire to protect and care for the family as a whole, even if that called for self-sacrifice [14]. They continuously create strategies to preserve the family structure and harmony [32], seeking to alleviate the children’s work overload [11,16].

About the most frequent coping strategies, the results of this study are congruent with the international findings, which highlight the importance of the grandparents striving to “live one day at a time” [11], accepting the illness, perseverance, optimism and the need for patience [14,16], believing [33,38], asking for spouse’s support [36] and the use of video calling to feel closer to their family [13].

In fact, the grandparents interviewed in this study express psychological distress and feel fragile both when confronted with the diagnosis and when adapting to the illness, which is also congruent with the research in this context [32]. Other authors, such as Wakefield and colleagues, also report that grandparents of children with cancer tend to experience greater distress, anxiety, depression, and anger, with clinical relevance [38].

The literature is consensual in concluding that grandparents’ emotional expression does not reflect the extent of their emotional pain, thus reinforcing the importance of bridging the lack of support resources available to this population [26]. In this regard, Findler also underlined that a higher level of stress is related to the perception of less legitimacy in expressing what they feel and experiencing personal growth [36].

Indeed, some authors report that grandparents feel more included and secure when they are involved in communicating about the grandchild’s health, which leads one to reflect on the circumstances in which children may omit information from their parents to maintain privacy and/or protect them from the impact of bad news [11].

Moreover, grandparents expressed fear of the future, which may be related to the implicit threat of the child/youth’s loss of life and the increased awareness of the illness-related risk [14]. The frustration underlying these emotional dimensions was also described in the case study by Kuhn and colleagues [11]. 

These findings, together with the fear of leaving the children helpless, are also in line with the international literature on grandparents’ future perspectives and their concern for quality of life in the future [14]. However, studies such as that of Trindade and colleagues concluded that grandparents can also feel optimism and enthusiasm, associated with their capacity for acceptance and gratitude [16].

In fact, following a traumatic event, individuals may experience improvement where it is possible to surpass what was present before the trauma [39]. Narratives are particularly important in this dimension, as the sharing of experiences and memories among the family supports the posttraumatic growth of all those involved [14].

Findler, Dayan-Sharabi and Yaniv found that grandparents of children who survived cancer showed more relief, pride in their contribution and greater family closeness [36]. Moreover, the literature reinforces the results of the present study, mentioning that there is a reinforcement of intra-family solidarity [40] and a greater learning and appreciation about the value of health and life [16].

Finally, the grandparents were found to value and appreciate the guidance from the health and school professionals, which reinforces the importance of grandparents’ communication with the multidisciplinary team and feeling accepted in their psychological distress [26].

The present study has some limitations, such as the small and non-probabilistic sample size, as well as data collection by telephone due to the difficulty in accessing this population. The diversity of complex chronic diseases that were included in the sample and the heterogeneity in the age of the sick children and youths—although important to include in a descriptive-exploratory study—are two factors that may also constitute limitations by dispersing the findings and making their generalization difficult. 

In turn, this research has the following strengths: recourse to the Portuguese population where there are not many studies focused on different family subsystems, giving attention to grandparents as an underexplored group in the literature and in clinical intervention, grandparents being the direct informants, data collection having been creative since the script was all thought and built for this study, meeting the needs of grandparents and collecting data through telephone calls, collecting comprehensive and diverse information about their psychological experience, the therapeutic effect of the qualitative methodology itself and the reach of 19 grandparents.

This study with its design seeks to pave the way for new lines of research, such as exploring the communication with health professionals, the perspective of parental caregivers on the contributions of grandparents to family dynamics, the perspective of healthy grandchildren about their grandparents’ role and the impact of intensive and end-of-life care. It would also be important to deepen the knowledge regarding protective factors in the adaptation to illness and, consequently, in the reduction of psychopathological symptoms. 

In addition, studies with larger samples and with a longitudinal nature that deepen the experience of other members of the family, such as healthy siblings, would be beneficial. It would also be interesting to study the perspective and experience of health professionals on the inclusion of grandparents in care provisions.

## 5. Conclusions

Grandparents play a central role within the family system, influencing and contributing significantly to intergenerational relationships. In the context of PPC, it is important to understand their role, responsibilities and challenges, with a view to elaborating responses to their needs and emotional well-being. 

This qualitative research covered several dimensions of the psychological experience of grandparents based on a sample consisting of direct informants, namely those with first-hand experience. Furthermore, the study explores different illnesses at different stages of evolution, with the aim of understanding and improving family relations and, ultimately, promoting sick child well-being.

The data collection was conducted in a creative manner, through a script with questions based on sharing a testimony with other grandparents and the principles of Narrative Therapy. From here, the results provide an integrated view on 10 essential dimensions in the psychological experience of grandparents. Anxiety, awareness of the unpredictability of the disease, the threat implicit in everyday life, fear of the future, the attempts to support the whole family and the need for opportunities for emotional expression—that does not reflect the extent of their emotional pain—are the main findings.

This research allowed for the identification of concrete support needs, as well as of important coping strategies and resources for this population, within a holistic and ecosystemic dynamic. The results translate into important contributions to raise the awareness of health professionals who come into contact with these families and the community as to the psychological experience of grandparents, constituting a significant source for psychoeducation. Furthermore, the findings translate into relevant information to research and systemic psychological intervention in the context of PPC. The development and implementation of support groups among grandparents is suggested to foster the sharing of experiences, the identification with other families in similar circumstances and, finally, the mutual help, which was also mentioned throughout this study. Peer support creates a shared social identity, fosters learning from others’ experiences, increases personal growth and develops helpful supportive communities.

Regarding perspectives for psychological intervention, the promotion of their coping strategies is also suggested, as well as the support in managing anxiety, the involvement of the different members of the family system and the enhancement of the marital dyad. In addition, it is essential to contribute to the participation of these grandparents in leisure moments and in opportunities for emotional expression. Finally, the communication with health care professionals is a point to consider and improve, allowing an adjustment between the grandparents’ needs and the multidisciplinary teams’ practices, contributing to reduce the feeling of exclusion from the clinical context.

Finally, this research enables the construction of specific intervention methodologies for this population. Although the field of pediatric palliative care is a relatively new and evolving specialty, one of the major challenges in psychology is to give support to the families in a holistic and ecosystemic dynamic, nurturing the importance of a psychological approach geared towards prevention and intervention with the whole family system.

## Data Availability

The data that support the findings of this study are available on request from the corresponding author. The data are not publicly available due to them containing information that could compromise the privacy of research participants.

## References

[1] Carter B.S., Levetown M., Friebert S.E. (2013). Palliative Care for Infants, Children and Adolescents—A Practical Handbook.

[2] Goldman A., Hain R., Liben S. (2012). Oxford Textbook of Palliative Care for Children.

[3] World Health Organization (1998). Cancer Pain Relief and Palliative Care in Children.

[4] International Children’s Palliative Care Network (ICPCN) (2023). Impact Report March 2023. https://icpcn.org/wp-content/uploads/2023/04/ICPCN-Impact-Report-FINAL.pdf.

[5] Observatório Português dos Cuidados Paliativos [OPCP] (2020). Relatório de Outono 2019.

[6] Together for Short Lives (TFSL) (2018). A Guide to Children’s Palliative Care.

[7] Feudtner C., Nye R.T., Boyden J.Y., Schwartz K.E., Korn E.R., Dewitt A.G., Waldman A.T., Schwartz L.A., Shen Y.A., Manocchia M. (2021). Association between children with life-threatening conditions and their parents’ and siblings’ mental and physical health. JAMA Netw. Open.

[8] Yagiela L.M., Hartman M.E. (2021). Family functioning when a child has a serious illness: All for one and one for all. JAMA Netw. Open.

[9] Moura M.J., Salazar H. (2017). A psicologia em cuidados paliativos pediátricos. Intervenção Psicológica em Cuidados Paliativos.

[10] Bronfenbrenner U. (1979). The Ecology of Human Development: Experiments by Nature and Design.

[11] Kuhn E., Schalley S., Potthoff M., Weaver M.S. (2019). The arc of generational care: A case series considering grandparent roles and care needs in pediatric palliative care. J. Soc. Work End Life Palliat. Care.

[12] Powdthavee N. (2011). Life Satisfaction and Grandparenthood: Evidence from a Nationwide Survey.

[13] Dunifon R., Near C., Ziol-Guest K. (2018). Back up parents, playmates, friends: Grandparents’ time with grandchildren. J. Marriage Fam..

[14] Miller E., Buys L., Woodbridge S. (2012). Impact of disability on families: Grandparents’ perspective. J. Intellect. Disabil. Res..

[15] Coall D.A., Hertwig R. (2010). Grandparental investment: Past, present, and future. Behav. Brain Sci..

[16] Trindade T.R., Hayashi M., Lourenço G., Figueiredo M., Silva C., Martinez C. (2020). Supports and relationships between mothers and grandparents of children with disability: Talking about intergenerational family solidarity. Cad. Bras. Ter. Ocup..

[17] Fingerman K.L. (2004). The role of offspring and in-laws in grandparents’ ties to their grandchildren. J. Fam. Issues.

[18] Jappens M., Van Bavel J. (2016). Parental divorce, residence arrangements, and contact between grandchildren and grandparents. J. Marriage Fam..

[19] Tan J.P. (2018). Do grandparents matter? Intergenerational relationships between the closest grandparents and Malaysian adolescents. J. Acad. Soc. Sci..

[20] Findler L. (2014). The experience of stress and personal growth among grandparents of children with and without intellectual disability. Intellect. Dev. Disabil..

[21] Flury M., Orellana-Rios C., Bergsträsser E., Becker G. (2020). “This is the worst that has happened to me in 86 years”: A qualitative study of the experiences of grandparents losing a grandchild due to a neurological or oncological disease. J. Spec. Pediatr. Nurs..

[22] Kelada L., Wakefield C.E., Doolan E.L., Drew D., Wiener L., Michel G., Cohn R.J. (2018). Grandparents of children with cancer: A controlled comparison of perceived family functioning. Support. Care Cancer.

[23] Arnone M., Dumond L.G., Yazdani N., Baroudi R.E., Pouliot A., Modanloo S. (2021). Evaluation of a grandparent bereavement support group in a Pediatric Palliative Care Hospice. Prog. Palliat. Care.

[24] Tatterton M.J., Walshe C. (2018). Understanding the bereavement experience of grandparents following the death of a grandchild from a life-limiting condition: A meta-ethnography. Lead. Glob. Nurs. Res..

[25] Pachalla S., Witting C., James K., Michelson K.N. (2020). Interventions for siblings, extended family, and community members after pediatric death. J. Loss Trauma.

[26] Lockton J., Oxlad M., Due C. (2021). Grandfathers’ experiences of grief and support following pregnancy loss or neonatal death of a grandchild. Qual. Health Res..

[27] Rusch R., Greenman J., Scanlon C., Horne K., Jonas D.F. (2020). Bibliotherapy and bereavement: Harnessing the power of reading to enhance family coping in pediatric palliative care. J. Soc. Work End-of-Life Palliat. Care.

[28] Braun V., Clarke V. (2006). Using thematic analysis in psychology. Qual. Res. Psychol..

[29] White M., Epston D. (1990). Narrative Means to Therapeutic Ends.

[30] White M., Morgan A. (2006). Narrative Therapy with Children and Their Families.

[31] Endstrand R.Z., Roll-Pettersson L., Allodi M.W., Hirvikoski T. (2019). Needs of grandparents of preschool-aged children with ASD in Sweden. J. Autism Dev. Disord..

[32] Moraes E.S., Mendes-Castillo A. (2018). The experience of grandparents of children hospitalized in Pediatric Intensive Care Unit. J. Sch. Nurs..

[33] Moules N.J., Laing C.M., McCaffrey G., Tapp D.M., Strother D. (2012). Grandparents’ experiences of childhood cancer, part I: Doubled and silenced. J. Pediatr. Oncol. Nurs..

[34] Moules N.J., McCaffrey G., Laing C.M., Tapp D.M., Strother D. (2012). Grandparents’ experiences of childhood cancer, part 2: The need of support. J. Pediatr. Oncol. Nurs..

[35] Ravindran V.P., Rempel G.R. (2010). Grandparents and siblings of children with congenital heart disease. J. Adv. Nurs..

[36] Findler L., Dayan-Sharabi M., Yaniv I. (2014). The overlooked side of the experience: Personal growth and quality of life among grandparents of children who survived cancer. J. Fam. Soc. Work.

[37] Luo Y., LaPierre T.A., Hughes M.E., Waite L.J. (2012). Grandparents providing care to grandchildren: A population-based study of continuity and change. J. Fam. Issues.

[38] Wakefield C.E., Drew D., Ellis S.J., Doolan E.L., McLoone J.K., Cohn R.J. (2014). Grandparents of children with cancer: A controlled study of distress, support, and barriers to care. Psycho-Oncology.

[39] Calhoun L.G., Tedeschi R.G., Calhoun L.G., Tedeschi R.G. (2014). The foundations of posttraumatic growth: An expanded framework. Handbook of Posttraumatic Growth: Research and Practice.

[40] Cabral I.E., Moraes J.R.M.M. (2015). Family caregivers articulating the social network of a child with special health care needs. Rev. Bras. Enferm..

